# A single nucleotide mutation in the dual-oxidase 2 (*DUOX2*) gene causes some of the panda's unique metabolic phenotypes

**DOI:** 10.1093/nsr/nwab125

**Published:** 2021-07-15

**Authors:** Agata M Rudolf, Qi Wu, Li Li, Jun Wang, Yi Huang, Jacques Togo, Christopher Liechti, Min Li, Chaoqun Niu, Yonggang Nie, Fuwen Wei, John R Speakman

**Affiliations:** State Key Laboratory of Molecular Development, Institute of Genetics and Developmental Biology, Chinese Academy of Sciences, Beijing 100101, China; Institute of Zoology, Chinese Academy of Sciences, Beijing 100101, China; Institute of Microbiology, Chinese Academy of Sciences, Beijing 100101, China; State Key Laboratory of Molecular Development, Institute of Genetics and Developmental Biology, Chinese Academy of Sciences, Beijing 100101, China; Institute of Microbiology, Chinese Academy of Sciences, Beijing 100101, China; State Key Laboratory of Molecular Development, Institute of Genetics and Developmental Biology, Chinese Academy of Sciences, Beijing 100101, China; State Key Laboratory of Molecular Development, Institute of Genetics and Developmental Biology, Chinese Academy of Sciences, Beijing 100101, China; Institute of Biological and Environmental Sciences, University of Aberdeen, Aberdeen AB24 2TZ, UK; State Key Laboratory of Molecular Development, Institute of Genetics and Developmental Biology, Chinese Academy of Sciences, Beijing 100101, China; State Key Laboratory of Molecular Development, Institute of Genetics and Developmental Biology, Chinese Academy of Sciences, Beijing 100101, China; Institute of Zoology, Chinese Academy of Sciences, Beijing 100101, China; Institute of Zoology, Chinese Academy of Sciences, Beijing 100101, China; Centre of Excellence for Animal Ecology and Genetics, Chinese Academy of Sciences, Kunming 650223, China; State Key Laboratory of Molecular Development, Institute of Genetics and Developmental Biology, Chinese Academy of Sciences, Beijing 100101, China; Institute of Biological and Environmental Sciences, University of Aberdeen, Aberdeen AB24 2TZ, UK; Centre of Excellence for Animal Ecology and Genetics, Chinese Academy of Sciences, Kunming 650223, China; Shenzhen Key Laboratory of Metabolic Health, Center for Energy Metabolism and Reproduction, Shenzhen Institute of Advanced Technology, Chinese Academy of Sciences, Shenzhen 518055, China

**Keywords:** giant panda, *DUOX2* mutation, metabolic rate, thyroid hormones, *DUOX2*, mice and microbiota

## Abstract

The giant panda (*Ailuropoda melanoleuca*) is an iconic bear native to China, famous for eating almost exclusively bamboo. This unusual dietary behavior for a carnivore is enabled by several key adaptations including low physical activity, reduced organ sizes and hypothyroidism leading to lowered energy expenditure. These adaptive phenotypes have been hypothesized to arise from a panda-unique single-nucleotide mutation in the dual-oxidase 2 (*DUOX2*) gene, involved in thyroid hormone synthesis. To test this hypothesis, we created genome-edited mice carrying the same point mutation as the panda and investigated its effect on metabolic phenotype. Homozygous mice were 27% smaller than heterozygous and wild-type ones, had 13% lower body mass-adjusted food intake, 55% decreased physical activity, lower mass of kidneys (11%) and brain (5%), lower serum thyroxine (T4: 36%), decreased absolute (12%) and mass-adjusted (5%) daily energy expenditure, and altered gut microbiota. Supplementation with T4 reversed the effects of the mutation. This work uses a state-of-the-art genome editing approach to demonstrate the link between a single-nucleotide mutation in a key endocrine-related gene and profound adaptive changes in the metabolic phenotype, with great importance in ecology and evolution.

## INTRODUCTION

A key aim in ecology is to understand the factors that underlie metabolic phenotypes and their ecological consequences, also known as the metabolic theory of ecology [1]. A parallel key goal in evolutionary ecology is to understand how genetic mutations build the phenotype that is then selected for in a given environment. However, linking differences in the metabolic phenotype to underlying genetic changes is challenging [2]. Most inferences come mainly from either phenotypic or genetic data alone, and studies rarely investigate associations between molecular evolution and adaptive changes in phenotypic traits [3]. The link between changes in a single gene and structural and functional phenotypic changes is difficult to demonstrate experimentally [3], especially in large protected species of animals, where laboratory manipulation of the gene is not an option. Mutations of key genes involved in endocrine function can have major phenotypic impacts [4,5]. However, the role of such mutations in species ecology and evolution is unclear.

The giant panda (*Ailuropoda melanoleuca)* is an iconic species of wildlife conservation, characterized by its unique biology. Pandas are bears that are endemic to China and feed almost exclusively on bamboo, which has very low nutritional value [6]. Feeding on this resource is only possible because of adaptations that have dramatically reduced panda energy demands, thereby reducing food intake requirements [7]. This low metabolism is enabled by extremely low levels of physical activity [7]. In nature they typically move <500 m daily and spend ∼40% of their time resting [6]. Moreover, pandas have smaller brains, kidneys and livers compared to other large mammals, which may also contribute to their low metabolic rates [7,8]. In addition, they have low levels of thyroid hormones thyroxine (T4) and triiodothyronine (T3), which average ∼50% and ∼60%, respectively, of that expected for similar-sized mammals [8]. These aspects of their unusual metabolic phenotype have been hypothesized to stem from a panda-unique single-nucleotide mutation in the *DUOX2* gene [7], which is absent in other carnivores, mice and humans. In pandas the mutation involves substitution of C with T, resulting in an Arginine to Termination codon in the 16th exon of the *DUOX2* gene. It is not yet known whether this premature stop codon results in no translation of the gene or whether a truncated version of the protein is produced that may have biological functions.

*DUOX2* encodes a protein involved in a critical step of thyroid hormone synthesis [9]. Some previously discovered mutations in *DUOX2*, or its maturation factor *DUOXA*, in mice [4,10] and humans [5,9,11–14] have been linked to goiter, dwarfism and congenital hypothyroidism. More generally, levels of thyroid hormones are associated with variations in metabolic rate [15–17] and physical activity [18,19]. DUOX2 is also involved in antimicrobial defense of the alimentary and respiratory tract [20,21]. However, the phenotypic consequences of the single-nucleotide panda-specific mutation remain unknown. Potentially, the *DUOX2* mutation may affect diverse aspects of physiology, particularly metabolic characteristics, which would then be involved in shaping wide aspects of panda ecology, including its bamboo diet, its behavior, reproduction and geographical distribution [22]. We aimed to test the hypothesis that aspects of the unusual giant panda metabolic phenotype can be traced to the single base pair mutation in the *DUOX2* gene. To identify the effects of the panda-specific mutation in this wild species, we used a genetically engineered (CRISPR-Cas9) mouse model carrying the same mutation as the one found in the giant panda (Fig. S1), i.e. substitution of C with T, 625 Arginine to Termination codon in the 15th exon of the mouse *DUOX2* gene. We investigated growth rate and traits associated with energy balance, such as metabolic rate, spontaneous physical activity and food consumption, body composition, energy assimilation, water consumption and mass of the vital organs, as well as circulating levels of thyroid hormones and composition of the fecal microbiota. We further demonstrate that the phenotypic consequences of this mutation do not stem from the microbiota changes, and can be reversed by supplementation with exogenous T4.

## RESULTS

**Figure 1.**
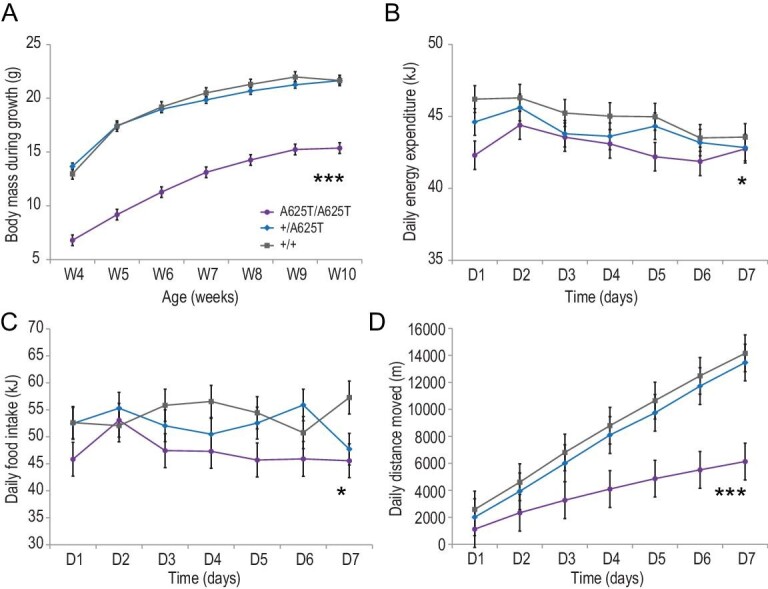
(A) Body mass between 4 and 10 weeks old (g/week), (B) body-mass-adjusted daily energy expenditure (kJ/day) and (C) daily food intake (kJ/day) in respirometry chamber, and (D) accumulated daily distance moved (m/day) in mutant homozygous (*Duox2*^A625T/A625T^), heterozygous (*Duox2*^+/A625T^) and wild-type homozygous (*Duox2*^+/+^) mice carrying a giant-panda-specific mutation in the *Duox2* gene; LSM with SE.

The panda-specific *Duox2* mutation in mice led to changes in phenotype that mirrored the unique biology of the panda. The *Duox2* mutant homozygote animals (*Duox2*^A625T/A625T^) were much smaller than heterozygous (*Duox2*^+/A625T^) and wild-type (*Duox2*^+/+^) animals between the ages of 4 and 10 weeks, i.e. during growth (mean body mass 12.2 (*Duox2*^A625T/A625T^) vs. 19.1 (*Duox2*^+/A625T^) and 19.3 (*Duox2*^+/+^) g SE ± 0.4, F_2,707 _= 534.79, P < 0.001; Fig. 1A, Tables S1 and S2). The *Duox2*^A625T/A625T^ mice were 27% dwarfed at 10–11 weeks old (16.0 (*Duox2*^A625T/A625T^), 21.4 (*Duox2*^+/A625T^), 21.9 (*Duox2*^+/+^) g SE ± 0.5, F_2,57 _= 53.77, P < 0.001; Tables S5 and S7), during which time we made physiological measurements. During seven days of measurements, the *Duox2*^A625T/A625T^ mice were on average 55% less active (moving 3903 (*Duox2*^A625T/A625T^) vs. 7851 (*Duox2*^+/A625T^) and 8579 (*Duox2*^+/+^) m SE ± 514, F_2,399 _= 23.92, P < 0.001; Fig. 1D, Tables S1 and S2), had lower daily energy intake by 25% (42.7 (*Duox2*^A625T/A625T^), 54.3 (*Duox2*^+/A625T^), 56.8 (*Duox2*^+/+^) kJ/day SE ± 1.1, F_2,397 _= 45.51, P < 0.001; Tables S1 and S2) and body mass-adjusted daily food intake was 13% lower (47.3 (*Duox2*^A625T/A625T^) vs. 52.3 (*Duox2*^+/A625T^) and 54.2 (*Duox2*^+/+^) kJ/day SE ± 1.4, F_2,396 _= 3.98, P = 0.020; Fig. 1C, Tables S1 and S2). The *Duox2*^A625T/A625T^ mice had a lower respiratory exchange ratio (0.78 (*Duox2*^A625T/A625T^) vs. 0.83 (*Duox2*^+/A625T^) and 0.82 (*Duox2*^+/+^) SE ± 0.01, F_2,399 _= 45.44, P < 0.001; Tables S1 and S2). The daily energy expenditure (DEE) was decreased in *Duox2*^A625T/A625T^ mice by 12% (40.6 (*Duox2*^A625T/A625T^), 44.9 (*Duox2*^+/A625T^), 46.3 (*Duox2*^+/+^) kJ/day SE ± 0.4, F_2,399 _= 70.02, P < 0.001), as well as daily resting energy expenditure (REE) by 10% (29.4 (*Duox2*^A625T/A625T^), 31.6 (*Duox2*^+/A625T^), 32.8 (*Duox2*^+/+^) kJ/day SE ± 0.4, F_2,399 _= 24.02, P < 0.001; Tables S1 and S2). These differences remained significant but were lower after adjustment for body size (5% for DEE: 42.9 (*Duox2*^A625T/A625T^) vs. 43.9 (*Duox2*^+/A625T^) and 44.9 (*Duox2*^+/+^) kJ/day SE ± 0.4, F_2,398 _= 4.13, P = 0.017; Fig. 1B, Table S1, and 6% for REE: 30.5 (*Duox2*^A625T/A625T^) vs. 31.2 (*Duox2*^+/A625T^) and 32.2 (*Duox2*^+/+^) kJ/day SE ± 0.4, F_2,398 _= 2.95, P = 0.053; Tables S1 and S2). The females were 14% smaller than males between 4–10 weeks old (16.6 females vs. 19.3 males g SE ± 0.2, F_1,714 _= 91.51, P < 0.001; Tables S3 and S4), and 14% smaller at 10–11 weeks old (18.3 vs. 21.3 g SE ± 0.5, F_1,58 _= 14.85, P < 0.001). The body mass-adjusted DEE (F_1,405 _= 0.13, P = 0.715), REE (F_1,405 _= 0.70, P = 0.403) or RER (F_1,406 _= 2.93, P = 0.088) did not differ between the sexes. However, females had 7% higher mass-adjusted food intake (53.1 vs. 49.5 kJ/day SE ± 1.0, F_1,403 _= 6.26, P = 0.013) and 28% higher activity (7606 vs. 5949 m SE ± 442, F_1,406 _= 7.02, P = 0.008) than males (Tables S3 and S4).

**Figure 2.**
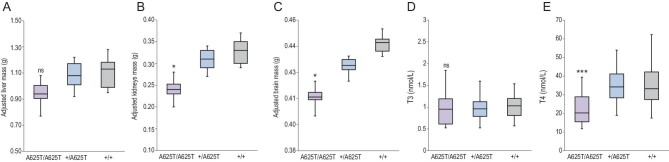
(A) Body-mass-adjusted liver, (B) kidneys and (C) brain mass, and (D) levels of T3-triiodothyronine and (E) T4-thyroxine (nmol/L) in mutant homozygous (*Duox2*^A625T/A625T^), heterozygous (*Duox2*^+/A625T^) and wild-type homozygous (*Duox2*^+/+^) mice carrying a giant-panda-specific mutation in the *Duox2* gene.

The average percent of lean mass corrected for body mass was higher in *Duox2*^A625T/A625T^ mice than in *Duox2*^+/A625T^ and *Duox2*^+/+^ mice (90.8% (*Duox2*^A625T/A625T^) vs. 87.8% (*Duox2*^+/A625T^) and 87.8% (*Duox2*^+/+^) SE ± 053, F_2,56 _= 3.53, P = 0.036), but the percent of fat was not significantly different (F_2,56 _= 2.05, P = 0.138; Tables S5 and S7). Adjusted daily water intake in metabolic cages was lower in *Duox2*^A625T/A625T^ mice (3.4 (*Duox2*^A625T/A625T^) vs. 6.4 (*Duox2*^+/A625T^) and 7.3 (*Duox2*^+/+^) g SE ± 0.5, F_2,53 _= 10.47, P < 0.001), and the daily urine production tended to be higher, but was not significant (2.1 (*Duox2*^A625T/A625T^), 1.3 (*Duox2*^+/A625T^), 1.4 (*Duox2*^+/+^) g SE ± 0.2, F_2,53 _= 2.87, P = 0.066; Tables S5 and S7). The energy assimilation did not differ between genotypes (90.9% (*Duox2*^A625T/A625T^), 90.8% (*Duox2*^+/A625T^), 90.9% (*Duox2*^+/+^) SE ± 0.2, F_2,57 _= 0.14, P = 0.873; Tables S5 and S7). After adjustment for body mass (organ masses, not dimensional or histological analysis), the *Duox2*^A625T/A625T^ mice had smaller kidneys (F_2,56 _= 4.44, P = 0.016; Fig. 2B), smaller brains (F_2,56 _= 4.70, P = 0.013; Fig. 2C) and spleens (F_2,56 _= 21.08, P < 0.001; Table S8) compared to *Duox2*^+/A625T^ and *Duox2*^+/+^, but adjusted liver size was not significantly different (F_2,56 _= 0.43, P = 0.652; Fig. 2A, Tables S6 and S8). Moreover, the *Duox2*^A625T/A625T^ mice tended to have smaller lungs (P = 0.061) and had smaller tails (P < 0.001), skin (P = 0.029), less mesenteric white adipose tissue (P < 0.001), but more subcutaneous white adipose tissue (P = 0.001) and bigger stomachs (P = 0.030; Tables S6 and S8). Masses of the other organs did not differ between the three genotypes (Tables S6 and S8).

**Figure 3.**
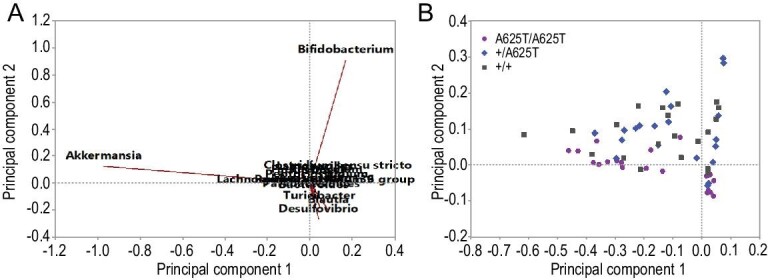
PCA analysis of fecal microbiota in mutant homozygous (*Duox2*^A625T/A625T^), heterozygous (*Duox2*^+/A625T^) and wild-type homozygous (*Duox2*^+/+^) mice carrying a giant-panda-specific mutation in the *Duox2* gene at the age of 10–11 weeks old: (A) PC1 and PC2 with microbiota genus; (B) PC1 and PC2 with genotype.

The level of serum T4 (thyroxine) was 36% lower in *Duox2*^A625T/A625T^ mice than in *Duox2*^+/A625T^ and *Duox2*^+/+^ mice (22.4 (*Duox2*^A625T/A625T^) vs. 34.3 (*Duox2*^+/A625T^) and 35.1 (*Duox2*^+/+^) nmol/L SE ± 2.2, F_2,56 _= 9.98, P < 0.001; Fig. 2E, Tables S5 and S7). However, serum T3 (triiodothyronine) did not differ between genotypes (F_2,56 _= 0.28, P = 0.757; Fig. 2D). Analyses of fecal microbiota revealed presence of 15 genera (Fig. 3A and Table S9). The analyses of scores from the principal component analysis (PCA) showed significant differences between genotypes (PC2: F_2,58 _= 13.48, P < 0.001; Tables S10 and S11), indicating that the *Duox2*^A625T/A625T^ mice had a lower abundance of commensal *Bifidobacterium* (correlation coefficient: 0.903) and slightly lower *Akkermansia* (0.123), but slightly higher *Desulfovibrio* (−0.268; Fig. 3B and Table S9).

To show if the effects of the *Duox2* mutation were a direct result of the mutation, or rather a consequence of changes in the gut microbiota, 4-week-old *Duox2*^+/+^ mice were exposed to an antibiotic mix in drinking water [23] and then gavaged with either *Duox2*^+/+^, *Duox2*^A625T/A625T^ or giant panda feces. The mice did not adopt the panda microbiome, but the microbiome from *Duox2*^+/+^- and *Duox2*^A625T/A625T^-treated mice was not distinct from the donors (Fig. S2A and B). In the *Duox2*^A625T/A625T^-treated mice, commensal *Akkermansia* were lower and *Lachnospiraceae* slightly higher (PC1: F_2,45 _= 3.94, P = 0.027; PC2: F_2,45 _= 4.66, P = 0.015; Fig. S2C and D, Tables S14–S16). The treatment did not affect the body mass (22.8 (*Duox2*^+/+^) vs. 22.7 (*Duox2*^A625T/A625T^) g SE ± 0.3, F_1,72 _= 0.01, P = 0.922), activity (2968 vs. 2772 m SE ± 177, F_1,72 _= 0.61, P = 0.438), mass-adjusted DEE (44.68 vs. 44.46 kJ/day SE ± 0.62, F_1,72 _= 0.061, P = 0.805) or REE (32.45 vs. 31.82 kJ/day SE ± 0.56, F_1,71 _= 0.65, P = 0.422), however, adjusted food intake (71.70 vs. 64.00 kJ/day SE ± 1.21, F_1,71 _= 20.17, P < 0.001) was lower in the mice with the *Duox2*^A625T/A625T^ microbiota (Tables S12 and S13).

**Figure 4.**
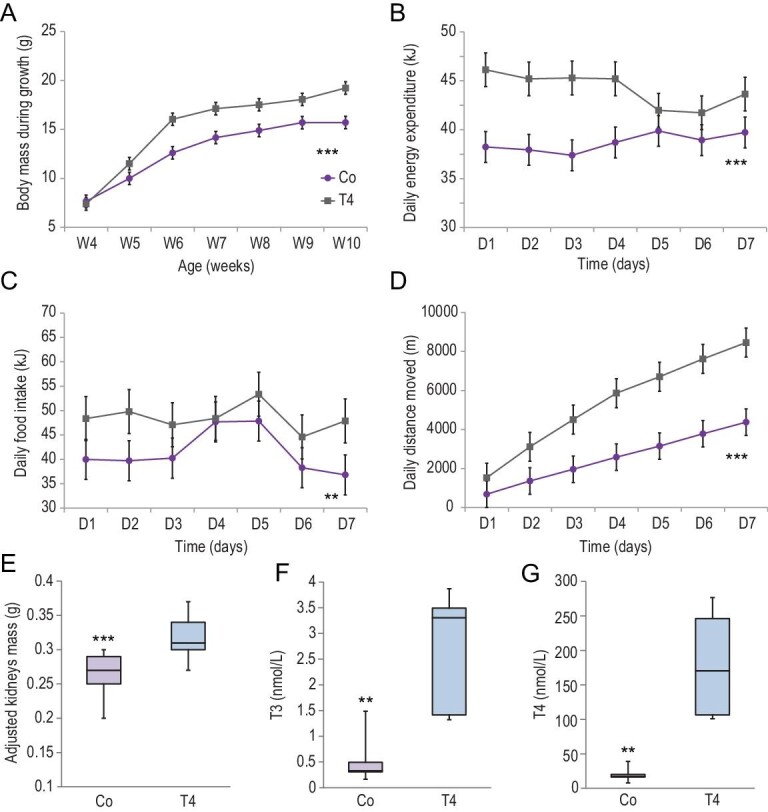
Reversal of the impact of the *Duox2* mutation by supplementation with T4. (A) Body mass between 4 and 10 weeks old (g/week), (B) body-mass-adjusted daily energy expenditure (kJ/day) and (C) daily food intake (kJ/day) in respirometry chamber, (D) accumulated daily distance moved (m/day), (E) body-mass-adjusted kidney mass and (F) levels of T3-triiodothyronine and (G) T4-thyroxine (nmol/L) in mutant homozygous (*Duox2*^A625T/A625T^) mice carrying a giant-panda-specific mutation in the *Duox2* gene; exposed (T4) and non-exposed (Co) to 0.5 µg/mL of T4 supplementation; LSM with SE.

To investigate if treatment with T4 would reverse the effects caused by the *Duox2* mutation, *Duox2*^A625T/A625T^ mice were exposed to 5 μg/mL of T4 in drinking water starting from 4 weeks old [24]. However, using this dose, serum T3 and T4 were increased by about 10 times above wild-type levels (Table S17), so we used a lower dose of 0.5 μg/mL T4, generating a more physiological effect. The T4 supplemented mice grew larger between 4 and 10 weeks old compared with un-supplemented mice (mean body mass 15.3 vs. 12.9 g SE ± 0.5, F_1,70 _= 46.06, P < 0.001; Fig. 4A, Tables S18 and S19). Moreover, they had higher activity by 53% (5400 vs. 2557 m SE ± 268, F_1,63 _= 56.07, P < 0.001; Fig. 4D) and higher adjusted food intake by 14% (48.49 vs. 41.51 kJ/day SE ± 1.70, F_1,62 _= 7.72, P = 0.007; Fig. 4C). Mass-adjusted DEE was also increased by 12% (44.17 vs. 38.69 kJ/day SE ± 0.65, F_1,62 _= 32.57, P < 0.001; Fig. 4B) and REE by 14% (30.89 vs. 26.53 kJ/day SE ± 0.69, F_1,62 _= 18.59, P < 0.001; Tables S18 and S19). The mass of their kidneys was higher (F_1,7 _= 73.55, P < 0.001; Fig. 4E) and they tended to have a higher percentage of fat (F_1,8 _= 5.12, P = 0.053; Tables S20 and S21). Under supplementation serum, thyroid hormones were both increased (T3: 1.8 vs. 0.4 SE ± 0.3, F_1,8 _= 12.81, P = 0.007; Fig. 4F and T4: 139.9 vs. 15.7 SE ± 19.8 nmol/L, F_1,8 _= 19.70, P = 0.002; Fig. 4G, Tables S20 and S21).

## DISCUSSION

In ecological and evolutionary studies, genotypic and phenotypic data have traditionally been investigated independently, which hinders identification of specific genetic mutations, the presence of which can be mechanistically linked to adaptive changes in the phenotype [3]. The phenotypic effects of single nucleotide mutations have been mainly inferred from predicted protein structures [2] or the evolution of simple organisms [25,26], where laboratory manipulation of the genes can be used to show the phenotypic changes, but are challenging to investigate in large protected animals. Here, we inferred the links between a single genotypic trait and multiple phenotypic characteristics relevant to a wild species of rare mammal with unique biology using a genetically manipulated laboratory mouse model. The genetic mutation we considered was a single nucleotide variant of a single gene involved in endocrine function, which led to disproportionately large changes to the metabolic and behavioral phenotype.

Among other hypothyroid mouse strains, a spontaneous mutation in *Duox2* has been reported to cause decreased body size and serum T4 levels [4]. Similar to this strain, the *Duox2*^A625T/A625T^ were also dwarfed, which in hypothyroid animals is a direct consequence of the effects of thyroid stimulating hormone (TSH) on bone and soft tissue growth, particularly effects on the hepatic expression of growth hormone receptor and consequent IGF1 production [4,27]. Moreover, thyroid hormones strongly influence neonatal growth especially in species with low maturity at birth, which includes rodents [27], but also pandas [28]. In fact, newborn panda offspring are, relatively, the smallest among all placental mammals [28], which seems a life history strategy to decrease the high energetic demands of reproduction. Consistently, our *Duox2*^A625T/A625T^ mice were much smaller than their littermates as pups, and they remained dwarfed as adults. Interestingly, in evolutionary history the giant panda seems to be dwarfed as well. The current giant panda, *A.melanoleuca,* evolved in the Holocene and shrunk in size relative to the extinct panda, *Ailuropoda baconi*, which was considerably larger and which was preceded by the small *Ailuropoda microta* [6]. However, the role of the *DUOX2* mutations in the evolution of panda body size remains unknown.

Our previous work indicated that giant pandas have lower than expected DEE [7], and the DEE and REE of the *Duox2*^A625T/A625T^ mice were also significantly decreased, even though the effect was reduced after correction for their small body mass. Therefore, it seems that the mutation in the *DUOX2* gene may have contributed to this unusual metabolic trait of the pandas. However, the impact of the mutation on DEE in mice was much lower than that observed in pandas [7]. Similar to pandas, the T4 level of the *Duox2*^A625T/A625T^ mice was decreased, but pandas also have lowered T3 levels, which was not observed in the *Duox2*^A625T/A625T^ mice. The mutation in *DUOX2* affects both T4 and T3 synthesis, but T3 is largely generated by conversion of T4 to T3 by deiodinases (DIO) [29]. We therefore explored whether this difference between the mice and pandas might be due to additional mutations in the panda DIO enzymes. We investigated amino acid sequences of DIO enzymes (Supplementary Data), but although we found some variation among different species, no pattern between the species with low and high T4 to T3 ratios was found (Fig. S3A–C). Therefore, mutations in *DIO* genes are unlikely to influence T4 to T3 conversion in the giant panda. Hence, in addition to the *DUOX2* mutation, the full metabolic phenotype of giant pandas likely depends on yet-unknown genetic mechanisms affecting T4 to T3 conversion, or TSH levels. Other possible mutations affecting panda metabolic phenotype may include genomic mitochondrial genes. Two such mutations have been identified in pandas, one in cytochrome c oxidase (*COX*), a rate-limiting enzyme of the electron transport chain [30], and another in *ATP8*, encoding and affecting post-translational modification of ATP synthase [31]. The effects caused by the *Duox2* mutation reported by Johnson *et al*. [4] appear larger than those observed in our mice, including a 90% decrease in T4 levels, versus a 36% decrease in *Duox2*^A625T/A625T^ mice. However, after recalculating the units used in both studies, the T4 of wild-type mice in the Johnson study is twice as high as those in our study. Also, the methodology used to assess T4 levels was different, as well as the age of the mice. Moreover, the T4 level in pandas is ∼50% of that expected for similar-sized mammals, which is close to the level we found. However, it is also possible that the panda-unique mutation leads to translation of a truncated protein, which still retains some biological functions.

Physical activity data for captive and wild pandas indicate that they spend only ∼30%–50% of their time on physical activity, which is lower than in other bears [7]. The difference in activity, i.e. daily distance moved, between our *Duox2*^A625T/A625T^ mice and the other two genotypes was very strong, which indicates that the *DUOX2* gene mutation may be largely responsible for the panda's low physical activity. The mutation may also affect the range of panda distribution, as pandas tend to choose parts of the forest with gentle slopes, allowing more energetically efficient travel [32]. The mutation also affected absolute and body-mass-corrected food intake. A reduction in physical activity, metabolic rate and food requirements would have been a major advantage to pandas when they started to consume the low-calorie bamboo, because it would have significantly reduced the amount of time per day that they would need to spend feeding. Hence, the *DUOX2* mutation may have been a key innovation in the panda lineage, enabling survival on their nutritionally poor bamboo diet.

In giant pandas, some organs are smaller compared to similar-sized mammals [7], and similarly, the kidneys and brains of *Duox2*^A625T/A625T^ mice were smaller. It is not known whether the spleen and lungs are also smaller in pandas, like in the *Duox2*^A625T/A625T^ mice. However, the vital organs account for ∼60% of resting energy expenditure, even though their weight is only ∼5% of total body mass [33]. Therefore, the low mass of the kidneys, brain and lungs probably contributed to the low metabolic rate of our *Duox2*^A625T/A625T^ mice, despite the liver mass being unchanged. The *Duox2*^A625T/A625T^ mice were overall leaner, and adipose tissue has low energy expenditure [33]. Brain size has previously been shown to be associated with physical activity, as several structural parts of the brain are enlarged in physically active humans [34], and consistently, brains of low-active *Duox2*^A625T/A625T^ mice and giant pandas are smaller. The smaller kidneys of *Duox2*^A625T/A625T^ mice seem to be associated with their increased urination. This may be due to retarded kidneys, or a direct effect of hypothyroidism leading to kidney injury [35]. Pandas also frequently urinate, which has been interpreted as social communication rather than a physiological dysfunction, but this may not be a correct interpretation. Despite the fact that they have a diet of bamboo, pandas have a carnivore's short digestive system, simple stomach and degenerated cecum [6], and although the *Duox2*^A625T/A625T^ mice had enlarged stomachs, the other sections of their gut remained unchanged.

The gut microbiota plays a critical role in health and physiology [36,37], and we observed that the *Duox2* mutation had a significant impact on the fecal microbiota. The fecal microbiota of *Duox2*^A625T/A625T^ mice was depleted in *Bifidobacterium* and *Akkermensia*. *Bifidobacterium* can be detected in adult pandas [21], but is especially abundant in panda infants [37], mirroring many animals. *Bifidobacterium* affects health, immunity and metabolism, due to its role in milk digestion and defense against pathogenic microbes [37]. In human adolescence, *Bifidobacterium* is gradually replaced by *Firmicutes/Bacteroidetes*. After panda cubs begin to eat bamboo the *Bifidobacterium* is replaced with other communities too. So, the *Duox2*^A625T/A625T^ mice microbiota was more similar to bamboo-eating adult pandas, yet not enriched with any cellulose-digesting genus, such as *Bacillus* or *Pseudomonas* [21]. However, as pandas and mice consume very different types of food, any extrapolation from mice to pandas should be done with caution. The *Duox2* expression is regulated by two signaling pathways, induced by normal gut microbiota, and is further upregulated in a state of dysbiosis [20,38]. Expression of *Duox2* and several other antimicrobial genes was increased in the gut epithelium of mice that were fed a low-protein diet, previously subjected to microbiota transplant from mice fed a high-fat diet [38]. Although *Bifidobacterium* and *Akkermensia* are not reported to induce *Duox2* expression as a defense mechanism of the gut [20,38], they have anti-inflammatory effects, and in *Duox2*^A625T/A625T^ mice we observed a mild increase of pathogenic *Desulfovibrio*. The microbiota of *Duox2*^+/+^ mice gavaged with microbiota from *Duox2*^A625T/A625T^ mice was poorer in *Akkermensia*, which, like *Bifidobacterium*, is common in healthy mice. This treatment led to decreased food intake. Thus, the *Duox2* mutation may have induced changes in the gut microbiome contributing to an overall reduction in food intake, but did not contribute to the other phenotypes observed.

Treatment with T4 at doses previously used in the literature [24,39] caused hyperthyroidism, but supplementation with the lower dose elevated serum T4 to a level that can be considered euthyroid [39]. The treatment also led to increased T3, likely because of its tissue conversion from T4 by DIO. Therefore, the treated mice did not completely resemble the wild-type mice. However, even so, we confirmed that by manipulating the T4 level, i.e. reversing the effect of the *Duox2* mutation on serum T4, the main phenotypic effects on growth, metabolism, activity, food intake and kidney mass were reversed and the magnitude of reversal almost exactly mirrored the deficit caused by the mutation. This confirms that physiological consequences of the single-nucleotide panda-specific mutation come almost exclusively from its effects on the endocrine system.

In summary, we demonstrated here that the decrease in T4 levels directly caused by the panda-unique mutation in the *Duox2* gene led to several changes in the metabolic phenotype, similar to those observed in the giant panda, and these changes were reversible by T4 supplementation. Hence, the mutation of this single nucleotide in a key endocrine gene resulted in disproportional changes to various adaptive physiological and anatomical traits. The mutation appears to underlie the low energetic requirements of the panda, which likely played an important role in the evolution of the panda-unique metabolic phenotype, affecting its ability to exploit bamboo, its life history strategy, aspects of its behavior and relation with its environment. Moreover, we demonstrated how links between genetic factors and adaptive changes in phenotypic characteristics of wild protected species can be investigated using laboratory model animals modified using state-of-the-art genome editing techniques.

## METHODS

All experimental procedures were approved by ethical committee numbers HP2018007, HP2019021 and HP2019030. To investigate the effects of a *Duox2* mutation, analogous to one discovered in the giant panda [7], a mouse model carrying this mutation (*Duox2*^+/A625T^ mice) has been genetically engineered using a conditional strategy CRISPR-Cas9. The mouse was based on the C57BL/6 background and contained heterozygous mutation to the *Duox2* gene, where the 625th Arginine was changed to a Termination codon (A625T). Next, the heterozygous population (*Duox2*^+/A625T^) was expanded to achieve the mutant homozygous (*Duox2*^A625T/A625T^) and wild-type homozygous (*Duox2*^+/+^) genotypes.

All newborn animals were genotyped before the experimental procedures using PCR. A balanced number of animals from both sexes and three genotypes was randomly assigned to the experiment. A series of anatomical and physiological traits were investigated in 10 male and 10 female individuals for each of the three genotypes: *Duox2*^+/+^, *Duox2*^+/A625T^ and *Duox2*^A625T/A625T^. To measure energy balance, the animals were maintained in individual cages and fed with baseline diet (D12450B, Research Diets, Inc.). After 1 week, the resting metabolic rate, spontaneous activity and food consumption were measured using the TSE PhenoMaster system (TSE PhenoMaster, Germany) for seven subsequent days. The body composition was measured before and after the metabolic rate measurement by magnetic resonance spectroscopy (EchoMRI-3N1-100^TM^). Next, energy assimilation, water consumption and urine production were measured in metabolic cages (Tecniplast, USA) for three days. Finally, the animals were killed by exposure to CO_2_ and dissected. The mass of various organs was measured and the blood was collected for analyses of thyroid hormones T4 and T3 levels. The analysis was performed using radioimmunoassay. The fecal samples were collected for analysis of the composition of microorganisms. The composition was investigated using DNA sequencing of the 16S rRNA gene.

To investigate if the effect of the *Duox2* mutation was a direct result of the mutation, or an indirect effect mediated by changes in gut microbiota, a subpopulation of juvenile *Duox2*^+/+^ mice were exposed to an antibiotics mix in the drinking water [23]. The gut microbiota was then repopulated by gavage with either *Duox2*^+/+^, *Duox2*^A625T/A625T^ or giant panda feces. Next, the metabolic rate, activity and food intake was measured and the fecal samples were analyzed.

To investigate if treatment with T4 would reverse the phenotypic effects caused by the *Duox2* mutation, a separate population of juvenile *Duox2*^A625T/A625T^ mice was exposed to T4 in drinking water. The animals were subjected to the same measurement protocols as the animals in the main part of the experiment. The experiment was initially performed using a 5 μg/mL concentration of T4 [24,39] and was later repeated on a different set of individuals using a 0.5 μg/mL concentration.

Statistical analyses were performed with IBM SPSS 23. One-way or two-way ANOVA and ANCOVA tests were used and results were presented as least square means (LSM) ± SE and P values at alpha 0.05. The fecal microbiota was analyzed with PCA, followed by one-way ANOVA.

A detailed description of the methods has been included in the Supplementary Data.

## DATA AVAILABILITY

The data underlying this article are available from the corresponding author John R. Speakman upon request, and are deposited in a public repository, Zenodo, accession number 10.5281/zenodo.4674583.

## Supplementary Material

nwab125_Supplemental_File
