# Supplementary material for: A single nucleotide mutation in the dual-oxidase 2 (DUOX2) gene causes some of the panda's unique metabolic phenotypes
Source: Natl Sci Rev. 2021 Jul 15;9(2):nwab125. doi: 10.1093/nsr/nwab125 (PMC8890364; doi:10.1093/nsr/nwab125)
Supplement: nwab125_Supplemental_File [file nwab125_supplemental_file.docx]

**Supplementary Materials**

**Title**

A single nucleotide mutation in the dual-oxidase 2 (*DUOX2*) gene causes some of the panda’s unique metabolic phenotypes

**Authors**

Agata M. Rudolf^1^, Qi Wu^2,3^, Li Li^1^, Jun Wang^3^, Yi Huang^1^, Jacques Togo^1^, Christopher Liechti^4^, Min Li^1^, Chaoqun Niu^1^, Yonggang Nie^2^, Fuwen Wei^2,5,6*^, John R. Speakman^1,4,5,*^

^1^ State key laboratory of molecular development, Institute of Genetics and Developmental Biology, Chinese Academy of Sciences, Beijing, China.

^2^ Institute of Zoology, Chinese Academy of Sciences, Beijing, China.

^3^ Institute of Microbiology, Chinese Academy of Sciences, Beijing, China.

^4^ Institute of Biological and Environmental Sciences, University of Aberdeen, Aberdeen, UK.

^5^ Centre of Excellence for Animal Ecology and Genetics, Chinese Academy of Sciences, Kunming, China.

^6^ Key Laboratory of Southwest China Wildlife Resources Conservation, China West Normal University, Nanchong, China.

* Corresponding authors

John R. Speakman: [j.speakman@abdn.ac.uk](mailto:j.speakman@abdn.ac.uk)

Fuwen Wei: weifw@ioz.ac.cn

**Materials and Methods**

All experimental procedures were approved by the ethical review board of the Institute of Genetics and Developmental Biology of the Chinese Academy of Sciences, approval numbers HP2018007, HP2019021 and HP2019030.

Mouse model

To investigate the effects of a DUOX2 mutation, analogous to one discovered in giant panda [7], a mouse model carrying this specific genetic mutation (DUOX2^+/A625T^ mice) has been genetically engineered using a conditional strategy CrispR-Cas9. The mouse was based on the C57BL/6 background, and contained heterozygous mutation to the DUOX2 gene at chromosome 2, exon 15, where CGA was changed to TGA, and therefore, 625^th^ Arginine was modified to a Termination codon (A625T). The premature stop codon next caused deletion of following regions among 32 exons present in mice DUOX2 gene, and if any protein was translated, resulted in its truncation. Next, the population of mice containing the heterozygous mutation (DUOX2^+/A625T^) was expanded using a cross-breeding strategy, to achieve also the mutant homozygous (DUOX2^A625T/A625T^) and wild-type homozygous (DUOX2^+/+^) genotypes. All litters were kept with the mother for four weeks after birth. Because the mutant homozygous offspring were very small, and unable to reach the water or food source, all pups were daily provided a wet food one week before and after weaning. Before the experiment, the animals were sustained in standard housing conditions, in specific pathogen free environment at 22-23^o^C and with water and standard rodent chow provided *ad libitum*.

Genotyping

All newborn animals were genotyped before the experimental procedures. DNA was extracted using the Tianamp Genomic DNA Kit (Tiangen Biotech Beijing, China) and amplified using PCR with specific primers designed to flank the targeted gene fragments. The PCR primers used for detection of the mutated fragments were designed to amplify a 990bp product (Forward 5`-3`; Reverse 5`-3`) and for the wild-type fragments to amplify a 516bp product from genomic DNA. The PCR products were electrophoresed on a 1.5% Agarose gel. We determined to which of the three genotypes each animal belonged, and then, a balanced number of animals from both sexes and three genotypes was randomly assigned to the experiment.

Phenotyping

A series of anatomical and physiological traits were investigated in 10 male and 10 female individuals for each of three genotypes: DUOX2^+/+^, DUOX2^+/A625T^, and DUOX2^A625T/A625T^. The animals were weighed weekly during growth, i.e. between the age of 4 and 10 weeks, and before and after the physiological measurements that took place in weeks 10-11.

To measure energy balance the animals were maintained in individual cages and for 2 weeks fed with baseline diet containing 10% fat, 20% protein and 70% carbohydrate with 35% sucrose (D12450B, Research Diets, Inc.). After one week of adjusting to the diet, the resting metabolic rate, spontaneous daily activity, and food consumption were measured using a TSE PhenoMaster system (TSE PhenoMaster, Germany). Each animal was weighed and placed inside the respirometry chamber cage, with *ad libitum* access to water, baseline food, and with a plastic swing for measuring body mass. Measurements for each individual were performed at a constant 22-23^o^C temperature, for seven subsequent days and nights, starting from 16:30 and finishing at 14:00. Briefly, metabolic rate was measured as the rate of oxygen consumption over 6 minute-long readings (VO_2_; ml O_2_/min). Fresh air dried with a TSE AirDrying unit (series 994620) was passed through the chamber at about 330 ml/min regulated by mass flow controller to 1.1% accuracy. Oxygen and carbon dioxide concentrations were measured in a sample of ~250 ml/min of excurrent air with TSE System CaloSys sensors (994620 series). The rates of O_2_ consumption, CO_2_ production and respiratory exchange ratio were recorded and calculated with TSE PhenoMaster Software V5.7.8. The resting metabolic rate was later calculated as lowest mean of four consecutive 6 minute (total 24 minutes) readings per each day. Daily energy expenditure (DEE) and resting energy expenditure (REE) were calculated for each measurement day separately from O_2_ consumption and CO_2_ production according to the equation: EE (kJ/day) = ((3.9 x VO2 (mL/min) + 1.1 x VCO2 (mL/min)) x 1440 (min)/1000 x 4.184 [40]. The mass of food consumed from the TSE feeders was weighed using TSE PermaSensors (259998 series) and the consumption was recorded with a DrinkingFeeding unit (series 259998). Daily food intake was calculated as follows: FI (kJ/day) = g food consumed daily*3.82(kcal/g food)*4.184 (energy density indicated by Research Diets manufacturer). The activity was measured with a set of TSE ActiMot2 sensor frames (series 303021) equipped with diodes emitting light beams. The system recorded horizontal X and Y level activity as well as vertical Z activity, such as rearing, via MoTil2 unit (series 302020) and provided a summary total activity data.

The body composition was measured before, and at the end of the metabolic rate measurement by magnetic resonance spectroscopy (Echo MRI model EchoMRI-3N1-100^TM^), which uses Quantitative Magnetic Resonance (QMR) technology to take direct measurements of total body fat and lean tissue mass, with Canola oil used as the standard for the measurements. Next, the animals were placed in metabolic cages (Tecniplast, USA), and their energy assimilation, daily water consumption and urine production were measured. The animals were tested for 3 days, during which they were fed with crushed baseline diet refilled at the second day of the trial, food and water were provided *ad libitum.* At the beginning and the end of the trial food, water, feces and urine were weighed with 0.01g accuracy. The energy absorption was calculated as: EA (%) = (1-(feces mass[g]/food intake[g]))*100.

The animals were killed by exposure to a high dose of CO_2_ concentration and dissected. The mass of various organs, such as empty sections of alimentary tract, liver, kidneys, heart, and brain was measured on a scale with 0.001g accuracy (Sartorius BSA223S-CW, Germany). The blood samples were collected directly from the heart for analyses of the thyroid hormones T4 and T3 (thyroxine and triiodothyronine) serum levels. The analysis was performed using RIA radioimmunoassay method with XH6080 radiometer (Reagents and analysis by Beijing North Biotechnology Research Institute Co., Ltd.). Briefly, the T4 or T3 concentration standards or the samples were added to a binding reagent to produce competitive immunological reaction with a sheep anti-T4 or anti-T3 antibody when incubated for 1 hour at 37 °C. After the binding moiety was separated from the free fraction using an anti-sheep immune separation reagent, the radioactivity of the bound portion was measured, and the corresponding binding rate was calculated. A standard curve was obtained by plotting the known T4 or T3 standard concentrations with the corresponding binding rate. The level of T4 or T3 in the tested samples was determined from the standard curve.

Finally, fecal samples from the colon were collected for analyses of the presence of various communities of microorganisms. The composition of microorganisms was investigated using DNA sequencing of the bacterial 16S rRNA gene. The 16S rRNA gene is ubiquitous in prokaryotic cells, and contains conserved and hypervariable regions. Conserved regions are used for amplification of the targeted fragment, while the analysis of hypervariable regions is used to identify bacterial species. The procedures were performed at Genomics Research Center, Institute of Microbiology, Chinese Academy of Sciences. Briefly, the microbial DNA samples were subjected to PCR amplification, library preparation and quantification of the designated region. Qualified libraries were sequenced using the Illumina Hiseq 2500 high throughput sequencing system (Illumina, Inc). Data were expressed in Operational Taxonomic Units (OTUs).

Manipulation of gut microbiota

To investigate to what extent the effect of the DUOX2 mutation was a direct result of the mutation, or an indirect effect mediated by changes in gut microbiota composition caused by the mutation, a subpopulation of 48 (males and females) four weeks-old DUOX2^+/+^ mice was used. Animals were exposed to a cocktail of antibiotics in the drinking water [23] for a week to clear out their original gut microbiota. The antibiotic mix consisted of ampicillin 0.5mg/ml, metronidazole 0.5mg/ml, gentamicin 0.5mg/ml, neomycin 0.5mg/ml, vancomycin 0.25mg/ml, and sucralose 4mg/ml. The gut microbiota was then repopulated by gavage with either DUOX2^+/+^, DUOX2^A625T/A625T^, or giant panda feces. Each of the donor material samples was a mix of feces collected from the same few individuals of various age and sex. The fecal material was placed in mice home cages, and dissolved to be either applied directly into the mouth (first three weeks of exposure, when animals were too small to gavage), or gavaged into the stomach (the following two weeks of treatment) in weekly intervals. Next, the animal’s metabolic rate, activity and food intake was measured for four consecutive days, and their fecal samples were collected, as well as the fecal samples from the donors. The composition of source and transplanted microbiota was analyzed with DNA sequencing of bacterial 16S rRNA as described above.

Thyroxine supplementation

To investigate if treatment with T4 would reverse some of the phenotypic effects caused by the DUOX2 mutation, a separate population of homozygous mutant mice (DUOX2^A625T/A625T^) was used. About six juvenile animals (balanced number of males and females) were exposed to T4 supplementation in concentration of 5 µg/mL [24,39] supplied *ad libitum* in drinking water, whereas another six were given just water. The water was prepared freshly every 3-4 days. The supplementation started at the age of 4 weeks, i.e. right after weaning, and lasted for 7 weeks. The animals were weighed weekly, and at the age of 10-11 weeks subjected to the same measurement protocols as animals in the main part of the experiment, described above. The experiment was later repeated in the same way, on a different set of individuals, using 0.5 µg/mL concentration of T4.

Statistical Analysis

Statistical analyses were performed with IBM SPSS 23 software. To analyse the data concerning the body mass at physiological measurements, energy assimilation and the level of thyroid hormones, one-way ANOVA was used, where the genotype was the main categorical factor with three levels (DUOX2^+/+^, DUOX2^+/A625T^, DUOX2^A625T/A625T^). For the analysis of fat and lean mass, water intake, urine production, and mass of organs, one-way ANCOVA was used, which included also body mass at the measurement as a fixed covariate. To analyse growth, metabolic rate, activity and food intake, ANOVA or ANCOVA were used, with the genotype, and the time factor with several levels corresponding to subsequent measurement weeks or days, as the main categorical factors. Body mass was used as a covariate in analyses of metabolic rate and food intake. The results were presented as least square means (LSM) ± SE, and P values at alpha 0.05. The fecal microbiota was analysed with PCA, and followed by ANOVA using the PCA scores.

**Amino acid alignment of DIO enzymes**

The DUOX2 gene affects both T4 and T3 synthesis, but T3 is largely generated by conversion of T4 to T3 by the DIO enzymes, mainly DIO2 [29]. Therefore, we explored whether this difference between the mice studied here, and the panda, might be due to additional mutations in the panda DIO2 enzyme. After investigating the amino acid sequence of DIO2 gene from 62 mammals, we found interspecific variation in the 92-109 AA region of human DIO2 (Fig S3.A). In humans, the 92th AA is Thr, but in carnivore species including the giant panda it is Ser, whereas the 105th AA in most species is Lys, but in the Ursidae family it is Thr. In humans, a Thr92Ala mutation reduces DIO2 activity and serum T3 levels [41], however, in humans Thr92Ser and Lys105Thr polymorphisms are unknown. Moreover, carnivores or bears other than pandas normally do not show decreased thyroid function, only in some hibernating bear species hypothyroidism was observed during this periodic fasting [42]. We compared the ratio of T4 to T3 in some species of carnivores, rodents, primates and ungulates (Fig S3.B). We found no clear pattern among the species with the same variants in the 92th and 105th position of DIO2. Because also DIO1 and DIO3 can be involved in T4 to T3 turnover, we compared amino acid alignment between species of carnivores with low and high T4 to T3 ratios, and in species from other taxa at 16 positions of the three DIO genes (Fig S3.C). Although we found some variation among the species with different T4 to T3 ratios, no clear pattern between the groups of species with low and high ratios could be concluded. Therefore, mutations in DIO genes are unlikely to influence T4 to T3 conversion in the giant panda.

**REFERENCES**

41. Castagna MG, Dentice M and Cantara S et al. DIO2 Thr92ala reduces deiodinase-2 activity and serum-T3 levels in thyroid-deficient patients. J Clin Endocrinol Metab 2017; 102: 1623–30.

42. Martinez B and Ortiz RM. Thyroid hormone regulation and insulin resistance: insights from animals naturally adapted to fasting. Physiology 2017; 32:141–51.

**Table S1.** Phenotypic impacts of homozygous, heterozygous and wild-type homozygous mice carrying Giant panda-specific mutation in DUOX2 gene: Results of repeated measures Mixed Model analyses: test statistics (F (df)) and significance levels (P).

|  | Fixed Effects | |  | |  |  |
| --- | --- | --- | --- | --- | --- | --- |
| Trait |  | Body mass | | Genotype | Time | Genotype*Time |
| BM 1 | F (df) |  | | 534.79 (2,707) | 144.46 (6,707) | 1.31 (12,707) |
|  | P |  | | **<0.001** | <0.001 | 0.205 |
| DEE adj | F (df) | 36.26 (1,398) | | 4.13 (2,398) | 2.10 (6,398) | 0.40 (12,398) |
|  | P | <0.001 | | **0.017** | 0.014 | 0.963 |
| DEE | F (df) |  | | 70.02 (2,399) | 2.48 (6,399) | 0.37 (12,399) |
|  | P |  | | **<0.001** | 0.023 | 0.974 |
| RER | F (df) |  | | 45.44 (2,399) | 7.39 (6,399) | 0.79 (12,399) |
|  | P |  | | **<0.001** | <0.001 | 0.665 |
| Act | F (df) |  | | 23.92 (2,399) | 18.44 (6,399) | 1.04 (12,399) |
|  | P |  | | **<0.001** | <0.001 | 0.415 |
| DFI adj | F (df) | 13.79 (1,396) | | 3.97 (2,396) | 0.45 (6,396) | 1.02 (12,396) |
|  | P | <0.001 | | **0.020** | 0.846 | 0.433 |
| DFI | F (df) |  | | 45.51 (2,397) | 0.44 (6,397) | 0.99 (12,397) |
|  | P |  | | **<0.001** | 0.855 | 0.462 |
| REE adj | F (df) | 8.50 (1,398) | | 2.95 (2,398) | 2.09 (6,398) | 0.65 (12,398) |
|  | P | 0.004 | | **0.053** | 0.053 | 0.799 |
| REE | F (df) |  | | 24.02 (2,399) | 2.06 (6,399) | 0.64 (12,399) |
|  | P |  | | **<0.001** | 0.058 | 0.810 |

BM 1 = body mass between the ages of 4 to 10 weeks-old [g]; DEE adj = body mass-adjusted daily energy expenditure [kJ/day]; DEE = daily energy expenditure [kJ/day]; RER = respiratory exchange ratio; Act = daily activity, distance moved [m/day]; DFI adj = body mass-adjusted daily food intake [kJ/day]; DFI = daily food intake [kJ/day]; REE adj = body mass-adjusted resting energy expenditure [kJ/day]; REE = resting energy expenditure [kJ/day].

**Table S2.** Adjusted means (LSM) of phenotypic traits from the Mixed Model analyses in mutant homozygous (DUOX2^A625T/A625T^), heterozygous (DUOX2^+/A625T^) and wild-type homozygous (DUOX2^+/+^) mice carrying Giant panda-specific mutation in the DUOX2 gene.

|  | Adjusted means: LSM (SE) | | |  |
| --- | --- | --- | --- | --- |
| Trait | Genotype | DUOX2^A625T/A625T^ | DUOX2^+/A625T^ | DUOX2^+/+^ |
| BM 1 |  | 12.18 (0.19) | 19.07 (0.12) | 19.29 (0.18) |
| DEE adj |  | 42.87 (0.52) | 43.99 (0.38) | 44.96 (0.41) |
| DEE |  | 40.56 (0.36) | 44.98 (0.36) | 46.28 (0.36) |
| RER |  | 0.78 (0.01) | 0.83 (0.01) | 0.82 (0.01) |
| Act |  | 3903 (514) | 7851 (514) | 8579 (514) |
| DFI adj |  | 47.23 (1.65) | 52.35 (1.21) | 54.22 (1.30) |
| DFI |  | 42.70 (1.12) | 54.29 (1.11) | 56.82 (1.12) |
| REE adj |  | 30.53 (0.53) | 31.15 (0.39) | 32.17 (0.42) |
| REE |  | 29.38 (0.36) | 31.65 (0.36) | 32.83 (0.36) |

BM 1 = body mass between the ages of 4 to 10 weeks-old [g]; DEE adj = body mass-adjusted daily energy expenditure [kJ/day]; DEE = daily energy expenditure [kJ/day]; RER = respiratory exchange ratio; Act = daily activity, distance moved [m/day]; DFI adj = body mass-adjusted daily food intake [kJ/day]; DFI = daily food intake [kJ/day]; REE adj = body mass-adjusted resting energy expenditure [kJ/day]; REE = resting energy expenditure [kJ/day].

**Table S3.** Phenotypic impacts of female and male mice carrying Giant panda-specific mutation in DUOX2 gene: Results of repeated measures Mixed Model analyses: test statistics (F (df)) and significance levels (P).

|  | Fixed Effects | |  | |  |  |
| --- | --- | --- | --- | --- | --- | --- |
| Trait |  | Body mass | | Sex | Time | Sex*Time |
| BM 1 | F (df) |  | | 91.52 (1,714) | 72.19 (6,714) | 1.02 (6,714) |
|  | P |  | | **<0.001** | <0.001 | 0.408 |
| DEE adj | F (df) | 139.14 (1,405) | | 0.13 (1,405) | 2.69 (6,405) | 0.71 (6,405) |
|  | P | <0.001 | | **0.715** | 0.014 | 0.642 |
| DEE | F (df) |  | | 30.34 (1,406) | 2.01 (6,406) | 0.53 (6,406) |
|  | P |  | | **<0.001** | 0.063 | 0.786 |
| RER | F (df) |  | | 2.93 (1,406) | 6.13 (6,406) | 0.88 (6,406) |
|  | P |  | | **0.088** | <0.001 | 0.506 |
| Act | F (df) |  | | 7.02 (1,406) | 16.65 (6,406) | 0.28 (6,406) |
|  | P |  | | **0.008** | <0.001 | 0.947 |
| DFI adj | F (df) | 99.59 (1,403) | | 6.26 (1,403) | 0.46 (6,403) | 0.59 (6,403) |
|  | P | <0.001 | | **0.013** | 0.841 | 0.735 |
| DFI | F (df) |  | | 4.30 (1,404) | 0.38 (6,404) | 0.51 (6,404) |
|  | P |  | | **0.039** | 0.894 | 0.803 |
| REE adj | F (df) | 36.43 (1,405) | | 0.70 (1,405) | 2.10 (6,405) | 1.20 (6,405) |
|  | P | <0.001 | | **0.403** | 0.052 | 0.308 |
| REE | F (df) |  | | 14.66 (1,406) | 1.93 (6,406) | 1.10 (6,406) |
|  | P |  | | **<0.001** | 0.075 | 0.362 |

BM 1 = body mass between the ages of 4 to 10 weeks-old [g]; DEE adj = body mass-adjusted daily energy expenditure [kJ/day]; DEE = daily energy expenditure [kJ/day]; RER = respiratory exchange ratio; Act = daily activity, distance moved [m/day]; DFI adj = body mass-adjusted daily food intake [kJ/day]; DFI = daily food intake [kJ/day]; REE adj = body mass-adjusted resting energy expenditure [kJ/day]; REE = resting energy expenditure [kJ/day].

**Table S4.** Adjusted means (LSM) of phenotypic traits from the Mixed Model analyses in female and male mice carrying Giant panda-specific mutation in the DUOX2 gene.

|  | Adjusted means: LSM (SE) | | |
| --- | --- | --- | --- |
| Trait | Sex | Females | Males |
| BM 1 |  | 16.64 (0.17) | 19.25 (0.22) |
| DEE adj |  | 43.86 (0.30) | 44.02 (0.30) |
| DEE |  | 42.67 (0.33) | 45.21 (0.33) |
| RER |  | 0.81 (0.00) | 0.82 (0.00) |
| Act |  | 7606 (442) | 5949 (442) |
| DFI adj |  | 53.06 (0.96) | 49.48 (0.96) |
| DFI |  | 49.80 (1.00) | 52.74 (1.00) |
| REE adj |  | 31.09 (0.31) | 31.46 (0.31) |
| REE |  | 30.47 (0.30) | 32.10 (0.30) |

BM 1 = body mass between the ages of 4 to 10 weeks-old [g]; DEE adj = body mass-adjusted daily energy expenditure [kJ/day]; DEE = daily energy expenditure [kJ/day]; RER = respiratory exchange ratio; Act = daily activity, distance moved [m/day]; DFI adj = body mass-adjusted daily food intake [kJ/day]; DFI = daily food intake [kJ/day]; REE adj = body mass-adjusted resting energy expenditure [kJ/day]; REE = resting energy expenditure [kJ/day].

**Table S5.** Phenotypic analysis of body composition in mutant homozygous, heterozygous and wild-type homozygous mice carrying Giant panda-specific mutation in DUOX2 gene: Mixed model test statistics (F (df)) and significance levels (P).

|  | Fixed Effects | |  | |
| --- | --- | --- | --- | --- |
| Trait | Body mass | | Genotype | |
|  | F (df) | P | F (df) | P |
| BM 2 |  |  | 53.77 (2,57) | **<0.001** |
| Fat mass | 7.74 (1,56) | 0.007 | 2.05 (2,56) | 0.138 |
| Lean mass | 19.53 (1,56) | <0.001 | 3.53 (2,56) | **0.036** |
| EnAssim |  |  | 0.14 (2,57) | 0.873 |
| DWI | 4.35 (1,53) | 0.042 | 10.47 (2,53) | **<0.001** |
| DUP | 0.08 (1,53) | 0.775 | 2.87 (2,53) | **0.066** |
| T3 |  |  | 0.28 (2,56) | 0.757 |
| T4 |  |  | 9.98 (2,56) | **<0.001** |

BM 2 = body mass at 10-11 weeks-old at physiological measurements [g]; Fat mass = body fat mass [%]; Lean mass = body lean mass [%]; EnAssim = energy assimilation [%]; DWI = daily water intake [g]; DUP = daily urine production [g]; T3 = triiodothyronine [nmol/L]; T4 = thyroxine [nmol/L].

**Table S6.** Results of Mixed Model analyses of organ mass in mutant homozygous, heterozygous and wild-type homozygous mice carrying Giant panda-specific mutation in DUOX2 gene: test statistics (F (df)) and significance levels (P).

|  | Fixed Effects | |  | |
| --- | --- | --- | --- | --- |
| Trait | Body mass | | Genotype | |
|  | F (df) | P | F (df) | P |
| Kidneys | 42.16 (1.56) | <0.001 | 4.44 (2,56) | **0.016** |
| Liver | 31.06 (1,56) | <0.001 | 0.43 (2,56) | 0.652 |
| Brain | 1.80 (1,56) | 0.185 | 4.70 (2,56) | **0.013** |
| Spleen | 0.24 (1,56) | 0.627 | 21.08 (2,56) | **<0.001** |
| Lungs | 6.83 (1,56) | 0.011 | 2.93 (2,56) | **0.061** |
| Sal Gla | 62.16 (1,56) | <0.001 | 1.78 (2,56) | 0.179 |
| MWAT | 13.96 (1,56) | <0.001 | 16.86 (2,56) | **<0.001** |
| SubWAT | 10.73 (1,55) | 0.002 | 7.55 (2,55) | **0.001** |
| Stomach | 3.85 (1,56) | 0.055 | 3.72 (2,56) | **0.030** |
| BAT | 7.08 (1,56) | 0.010 | 0.39 (2,56) | 0.678 |
| EpWAT | 73.85 (1,56) | <0.001 | 1.97 (2,56) | 0.147 |
| RtWAT | 0.51 (1,56) | 0.479 | 0.16 (2,56) | 0.849 |
| Heart | 21.84 (1,56) | <0.001 | 0.36 (2,56) | 0.717 |
| Pancreas | 0.23 (1,56) | 0.634 | 1.11 (2,56) | 0.338 |
| Small Int | 2.55 (1,56) | 0.116 | 1.89 (2,56) | 0.160 |
| Large Int | 0.026 (1,56) | 0.873 | 0.149 (2,56) | 0.862 |
| Caecum | 0.83 (1,56) | 0.365 | 1.13 (2,56) | 0.330 |
| Skin | 82.57 (1,55) | <0.001 | 3.77 (2,55) | **0.029** |
| Tail | 10.82 (1,56) | 0.002 | 13.83 (2,56) | **<0.001** |
| F Rep Org | 0.28 (1,26) | 0.600 | 0.43 (2,26) | 0.654 |
| M Rep Org | 1.90 (1,26) | 0.179 | 2.33 (2,26) | 0.117 |
| M Foll Gla | 0.01 (1,26) | 0.942 | 1.41 (2,26) | 0.262 |

Kidneys = mass of kidneys; Liver; Brain; Spleen; Lungs; Sal Gla = salivary gland; MWAT = mesenteric white adipose tissue; SubWAT = subcutaneous white adipose tissue; Lungs; Stomach; BAT = brown adipose tissue; EpWAT = epididymal white adipose tissue; RtWAT = retroperitoneal white adipose tissue; Heart; Pancreas; Small Int = small intestine; Large Int = large intestine; Caecum; Skin; Tail [g]; F = female; M = male; Rep Org = reproductive organs; Foll Gla = follicular gland [g].

**Table S7.** Adjusted means (LSM) for body composition from Mixed Model analyses in mutant homozygous (DUOX2^A625T/A625T^), heterozygous (DUOX2^+/A625T^) and wild-type homozygous (DUOX2^+/+^) mice carrying Giant panda-specific mutation in DUOX2 gene.

|  | Adjusted means: LSM (SE) | | |  |
| --- | --- | --- | --- | --- |
| Trait | Genotype | DUOX2^A625T/A625T^ | DUOX2^+/A625T^ | DUOX2^+/+^ |
| BM 2 |  | 16.03 (0.45) | 21.40 (0.45) | 21.94 (0.45) |
| Fat mass |  | 4.70 (0.61) | 6.45 (0.45) | 6.13 (0.48) |
| Lean mass |  | 90.09 (0.63) | 87.75 (0.47) | 87.81 (0.50) |
| EnAssim |  | 90.93 (0.20) | 90.78 (0.20) | 90.88 (0.20) |
| DWI |  | 3.44 (0.56) | 6.35 (0.45) | 7.29 (0.48) |
| DUP |  | 2.10 (0.23) | 1.30 (0.19) | 1.37 (0.20) |
| T3 |  | 0.97 (0.07) | 0.96 (0.07) | 1.03 (0.08) |
| T4 |  | 22.44 (2.23) | 34.29 (2.23) | 35.11 (2.29) |

BM 2 = body mass at 10-11 weeks-old at physiological measurements [g]; Fat mass = body fat mass [%]; Lean mass = body lean mass [%]; EnAssim = energy assimilation [%]; DWI = daily water intake [g]; DUP = daily urine production [g]; T3 = triiodothyronine [nmol/L]; T4 = thyroxine [nmol/L].

**Table S8.** Adjusted means (LSM) of organ mass in mutant homozygous (DUOX2^A625T/A625T^), heterozygous (DUOX2^+/A625T^) and wild-type homozygous (DUOX2^+/+^) mice carrying Giant panda-specific mutation in DUOX2 gene.

|  | Adjusted means: LSM (SE) | | |  |
| --- | --- | --- | --- | --- |
| Trait | Genotype | DUOX2^A625T/A625T^ | DUOX2^+/A625T^ | DUOX2^+/+^ |
| Kidneys |  | 0.274 (0.008) | 0.295 (0.007) | 0.308 (0.007) |
| Liver |  | 1.075 (0.037) | 1.028 (0.030) | 1.032 (0.031) |
| Brain |  | 0.416 (0.005) | 0.427 (0.004) | 0.439 (0.005) |
| Spleen |  | 0.050 (0.004) | 0.075 (0.003) | 0.085 (0.003) |
| Lungs |  | 0.138 (0.007) | 0.160 (0.005) | 0.160 (0.006) |
| Sal Gla |  | 0.110 (0.007) | 0.128 (0.006) | 0.123 (0.006) |
| MWAT |  | 0.160 (0.011) | 0.252 (0.009) | 0.223 (0.009) |
| SubWAT |  | 0.209 (0.019) | 0.112 (0.016) | 0.105 (0.016) |
| Stomach |  | 0.152 (0.005) | 0.142 (0.004) | 0.134 (0.004) |
| BAT |  | 0.092 (0.007) | 0.099 (0.005) | 0.100 (0.006) |
| EpWAT |  | 0.226 (0.017) | 0.207 (0.014) | 0.181 (0.014) |
| RtWAT |  | 0.040 (0.024) | 0.025 (0.020) | 0.038 (0.020) |
| Heart |  | 0.129 (0.005) | 0.132 (0.004) | 0.134 (0.004) |
| Pancreas |  | 0.107 (0.010) | 0.114 (0.008) | 0.127 (0.009) |
| Small Int |  | 0.671 (0.030) | 0.704 (0.025) | 0.751 (0.026) |
| Large Int |  | 0.147 (0.009) | 0.142 (0.007) | 0.147 (0.008) |
| Caecum |  | 0.114 (0.008) | 0.098 (0.006) | 0.098 (0.006) |
| Skin |  | 3.151 (0.081) | 3.465 (0.067) | 3.339 (0.070) |
| Tail |  | 0.384 (0.020) | 0.526 (0.016) | 0.517 (0.017) |
| F Rep Org |  | 0.104 (0.022) | 0.129 (0.017) | 0.114 (0.017) |
| M Rep Org |  | 0.237 (0.021) | 0.175 (0.012) | 0.171 (0.014) |
| M Foll Gla |  | 0.121 (0.037) | 0.206 (0.022) | 0.194 (0.025) |

Kidneys = mass of kidneys; Liver; Brain; Spleen; Lungs; Sal Gla = salivary gland; MWAT = mesenteric white adipose tissue; SubWAT = subcutaneous white adipose tissue; Lungs; Stomach; BAT = brown adipose tissue; EpWAT = epididymal white adipose tissue; RtWAT = retroperitoneal white adipose tissue; Heart; Pancreas; Small Int = small intestine; Large Int = large intestine; Caecum; Skin; Tail [g]; F = female; M = male; Rep Org = reproductive organs; Foll Gla = follicular gland [g].

**Table S9.** Results of Principal Component Analyses of feces microbiota in mutant homozygous, heterozygous and wild-type homozygous mice carrying Giant panda-specific mutation in DUOX2 gene: variance explained by PC and correlation coefficients for variables.

|  | PC1 | PC2 | PC3 |
| --- | --- | --- | --- |
|  | Proportion of variance explained | | |
| Cumulative | 0.648 | 0.803 | 0.845 |
|  | Correlation coefficient | | |
| Akkermansia | **-0.969** | **0.123** | 0.001 |
| Desulfovibrio | 0.040 | **-0.268** | 0.516 |
| Bifidobacterium | **0.168** | **0.903** | 0.195 |
| Lactobacillus | 0.023 | 0.042 | -0.588 |
| Blautia | 0.086 | -0.202 | 0.225 |
| Clostridium | 0.132 | 0.050 | -0.379 |
| Bacteroides | 0.019 | -0.090 | -0.103 |
| Parabacteroides | 0.007 | -0.082 | -0.142 |
| Faecalibaculum | 0.033 | 0.023 | 0.010 |
| Turicibacter | 0.045 | -0.173 | -0.254 |
| Peptoclostridium | 0.031 | -0.011 | 0.002 |
| Alistipes | 0.017 | 0.016 | 0.078 |
| Ruminiclostridium | 0.020 | -0.053 | 0.059 |
| Mucispirillum | 0.010 | -0.042 | 0.223 |
| Lachnospiraceae | 0.026 | -0.054 | 0.045 |

Variables = microorganism taxa: genus

**Table S10.** Results of Mixed Model analyses of feces microbiota (PC1, PC2 and PC3 scores from Principal Component Analysis) in mutant homozygous, heterozygous and wild-type homozygous mice carrying Giant panda-specific mutation in DUOX2 gene: test statistics (F (df)) and significance levels (P).

|  | Fixed Effects | |  |  |
| --- | --- | --- | --- | --- |
| Trait |  | PC1 | PC2 | PC3 |
| Genotype | F (df) | 0.90 (2,58) | 13.48 (2,58) | 0.33 (2,58) |
|  | P | 0.413 | **<0.001** | 0.723 |

PC1= Principal Component 1; PC2= Principal Component 2; PC3= Principal Component 3; scores from PC Analysis.

**Table S11.** Adjusted means (LSM) from Mixed Model analyses of feces microbiota (scores from Principal Component Analysis) in mutant homozygous (DUOX2^A625T/A625T^), heterozygous (DUOX2^+/A625T^) and wild-type homozygous (DUOX2^+/+^) mice carrying Giant panda-specific mutation in DUOX2 gene.

|  | Adjusted means: LSM (SE) | | |  |
| --- | --- | --- | --- | --- |
| Trait | Genotype | DUOX2^A625T/A625T^ | DUOX2^+/A625T^ | DUOX2^+/+^ |
| PC1 |  | -0.169 (0.037) | -0.099 (0.038) | -0.146 (0.038) |
| PC2 |  | -0.009 (0.015) | 0.093 (0.016) | 0.084 (0.016) |
| PC3 |  | 0.015 (0.010) | 0.026 (0.010) | 0.021 (0.010) |

PC1 = Principal Component 1; PC2 = Principal Component 2; PC3 = Principal Component 3; scores from PC Analysis.

**Table S12.** Results of Mixed Model analyses in wild-type mice exposed to microbiota from DUOX2^A625T/A625T^ or DUOX2^+/+^ donors: test statistics (F (df)) and significance levels (P).

|  | Fixed Effects | |  | |  |  |
| --- | --- | --- | --- | --- | --- | --- |
| Trait |  | Body mass | | Treatment | Time | Treatment*Time |
| BM | F (df) |  | | 0.01 (1,18) |  |  |
|  | P |  | | 0.961 |  |  |
| DEE adj | F (df) | 6.47 (1,71) | | 0.061 (1,71) | 1.28 (3,71) | 0.12 (3,71) |
|  | P | 0.013 | | 0.805 | 0.288 | 0.945 |
| DEE | F (df) |  | | 0.04 (1,72) | 1.19 (3,72) | 0.12 (3,72) |
|  | P |  | | 0.834 | 0.320 | 0.951 |
| RER | F (df) |  | | 6.41 (1,72) | 1.94 (3,72) | 0.60 (3,72) |
|  | P |  | | **0.014** | 0.131 | 0.619 |
| Act | F (df) |  | | 0.61 (1,72) | 32.48 (3,72) | 0.11 (3,72) |
|  | P |  | | 0.438 | <0.001 | 0.951 |
| DFI adj | F (df) | 8.64 (1,71) | | 20.17 (1,71) | 4.16 (3,71) | 055 (3,71) |
|  | P | 0.004 | | **<0.001** | 0.009 | 0.647 |
| DFI | F (df) |  | | 18.51 (1,72) | 3.76 (3,72) | 0.14 (3.72) |
|  | P |  | | **<0.001** | 0.014 | 0.683 |
| REE adj | F (df) | 6.55 (1,71) | | 0.65 (1,71) | 1.93 (3,72) | 0.01 (3.72) |
|  | P | 0.013 | | 0.422 | 0.132 | 0.999 |
| REE | F (df) |  | | 0.56 (1,72) | 1.79 (3,72) | 0.01 (3,72) |
|  | P |  | | 0.456 | 0.156 | 0.999 |

BM = body mass at the age of 10-11 weeks-old [g]; DEE adj = body mass-adjusted daily energy expenditure [kJ/day]; DEE = daily energy expenditure [kJ/day]; RER = respiratory exchange ratio; Act = daily activity, distance moved [m/day]; DFI adj = body mass-adjusted daily food intake [kJ/day]; DFI = daily food intake [kJ/day]; REE adj = body mass-adjusted resting energy expenditure [kJ/day]; REE = resting energy expenditure [kJ/day].

**Table S13.** Adjusted means (LSM) from Mixed Model analyses in wild-type mice exposed to microbiota from DUOX2^A625T/A625T^ or DUOX2^+/+^ donors.

|  | Adjusted means: LSM (SE) | |  | Adjusted means: LSM (SE) | |
| --- | --- | --- | --- | --- | --- |
| Trait | DUOX2^A625T/A625T^ | DUOX2^+/+^ | Trait | DUOX2^A625T/A625T^ | DUOX2^+/+^ |
| BM | 22.71 (0.63) | 22.75 (0.69) |  |  |  |
| DEE adj | 44.46 (0.59) | 44.68 (0.65) | DEE | 44.47 (0.61) | 44.66 (0.67) |
| RER | 0.84 (0.01) | 0.85 (0.01) | Act | 2772 (168) | 2968 (186) |
| DFI adj | 64.00 (1.15) | 71.70 (1.27) | DFI | 63.98 (1.21) | 71.73 (1.34) |
| REE adj | 31.82 (0.53) | 32.45 (0.58) | REE | 31.83 (0.55) | 32.44 (0.60) |

BM = body mass at the age of 10-11 weeks-old [g]; DEE adj = body mass-adjusted daily energy expenditure [kJ/day]; DEE = daily energy expenditure [kJ/day]; RER = respiratory exchange ratio; Act = daily activity, distance moved [m/day]; DFI adj = body mass-adjusted daily food intake [kJ/day]; DFI = daily food intake [kJ/day]; REE adj = body mass-adjusted resting energy expenditure [kJ/day]; REE = resting energy expenditure [kJ/day].

**Table S14.** Results of Principal Component Analyses of feces microbiota in wild-type mice exposed to microbiota from DUOX2^A625T/A625T^ or DUOX2^+/+^ donors: variance explained by PC and correlation coefficients for variables.

|  | PC1 | PC2 |
| --- | --- | --- |
|  | Variation explained | |
| Cumulative | 0.881 | 0.951 |
|  | Correlation coefficient | |
| Akkermansia | **-0.751** | **-0.589** |
| Flavonifractor | 0.009 | 0.131 |
| Oscillibacter | 0.019 | 0.208 |
| Unclassified Clostridiaceae | 0.050 | 0.123 |
| Unclassified Clostridiales | 0.013 | 0.089 |
| Unclassified Lachnospiraceae | **0.654** | **-0.718** |
| Unclassified Rhodospirillaceae | -0.026 | 0.070 |
| Unclassified Ruminococcaceae | 0.076 | 0.222 |

Variables = microorganism taxa: genus

**Table S15.** Results of Mixed Model analyses of feces microbiota (PC1 and PC2 scores from Principal Component Analysis) in wild-type homozygous mice exposed to microbiota from DUOX2^A625T/A625T^ or DUOX2^+/+^ mice or giant panda donors: test statistics (F (df)) and significance levels (P).

|  | Fixed Effects | |  |
| --- | --- | --- | --- |
| Trait |  | PC1 | PC2 |
| Treatment | F (df) | 3.94 (2,45) | 4.66 (2,45) |
|  | P | **0.027** | **0.015** |

PC1= Principal Component 1; PC2= Principal Component 2; scores from PC Analysis.

**Table S16.** Adjusted means (LSM) from Mixed Model analyses of feces microbiota (scores from Principal Component Analysis) in wild-type homozygous mice exposed to microbiota from DUOX2^A625T/A625T^ or DUOX2^+/+^ mice or giant panda (gp) donors.

|  | | Adjusted means: LSM (SE) | | |
| --- | --- | --- | --- | --- |
| Treatment | Trait | | PC1 | PC2 |
| DUOX2A625T/A625T |  | | 0.350 (0.137) | -0.424 (0.042) |
| DUOX2+/+ |  | | 0.204 (0.163) | -0.455 (0.057) |
| gp |  | | 0.188 (0.220) | -0.478 (0.046) |

PC1 = Principal Component 1; PC2 = Principal Component 2; PC3 = Principal Component 3; scores from PC Analysis.

**Table S17.** Levels of thyroid hormones in serum of mice given T4 in the drinking water: Results in mutant homozygous mice carrying Giant panda-specific mutation in DUOX2 gene exposed to **5 µg T4 /mL** drinking water: Mixed model test statistics (F (df)) and significance levels (P).

|  | Fixed Effects | |  | |
| --- | --- | --- | --- | --- |
| Trait | Treatment | | Adjusted means: LSM (SE) | |
|  | F (df) | P | T4 | Control |
| T3 | 278.08 (1,8) | **<0.001** | 7.14 (0.29) | 0.37 (0.29) |
| T4 | 215.54 (1,8) | **<0.001** | 480.70 (22.39) | 15.65 (22.39) |

T3 = triiodothyronine [nmol/L]; T4 = thyroxine [nmol/L].

**Table S18.** Results in mutant homozygous mice carrying Giant panda-specific mutation in DUOX2 gene exposed to **0.5 µg T4 /mL** drinking water: Mixed model test statistics (F (df)) and significance levels (P).

|  | Fixed Effects | |  | |  |  |
| --- | --- | --- | --- | --- | --- | --- |
| Trait |  | Body mass | | Treatment | Time | Treatment*Time |
| BM 1 | F (df) |  | | 46.06 (1,70) | 66.26 (6,70) | 2.17 (6,70) |
|  | P |  | | **<0.001** | <0.001 | 0.056 |
| DEE adj | F (df) | 23.48 (1,62) | | 32.57 (1,62) | 0.29 (6,62) | 1.15 (6,62) |
|  | P | <0.001 | | **<0.001** | 0.937 | 0.347 |
| DEE | F (df) |  | | 52.65 (1,63) | 0.22 (6,63) | 0.84 (6,63) |
|  | P |  | | **<0.001** | 0.970 | 0.541 |
| RER | F (df) |  | | 4.58 (1,63) | 0.51 (6,63) | 0.39 (6,63) |
|  | P |  | | **0.036** | 0.798 | 0.881 |
| Act | F (df) |  | | 56.07 (1,63) | 14.34 (6,63) | 1.40 (6,63) |
|  | P |  | | **<0.001** | <0.001 | 0.228 |
| DFI adj | F (df) | 12.77 (1,62) | | 7.72 (1,62) | 1.14 (6,62) | 0.32 (6,62) |
|  | P | 0.001 | | **0.007** | 0.350 | 0.925 |
| DFI | F (df) |  | | 18.33 (1,63) | 0.96 (6,63) | 0.27 (6,63) |
|  | P |  | | **<0.001** | 0.459 | 0.949 |
| REE adj | F (df) | 6.69 (1,62) | | 18.59 (1,62) | 0.10 (6,62) | 0.31 (6,62) |
|  | P | 0.012 | | **<0.001** | 0.996 | 0.930 |
| REE | F (df) |  | | 31.93 (1,63) | 0.10 (6,63) | 0.28 (6.63) |
|  | P |  | | **<0.001** | 0.997 | 0.943 |

BM 1 = body mass between the ages of 4 to 10 weeks-old [g]; DEE adj = body mass-adjusted daily energy expenditure [kJ/day]; DEE = daily energy expenditure [kJ/day]; RER = respiratory exchange ratio; Act = daily activity, distance moved [m/day]; DFI adj = body mass-adjusted daily food intake [kJ/day]; DFI = daily food intake [kJ/day]; REE adj = body mass-adjusted resting energy expenditure [kJ/day]; REE = resting energy expenditure [kJ/day].

**Table S19.** Effects of T4 supplementation: Adjusted means (LSM) from Mixed Model analyses in mutant homozygous mice carrying Giant panda-specific mutation in DUOX2 gene exposed (T4) and non-exposed (Control) to **0.5 µg T4 /mL** drinking water.

|  | Adjusted means: LSM (SE) | |  | Adjusted means: LSM (SE) | |
| --- | --- | --- | --- | --- | --- |
| Trait | T4 | Control | Trait | T4 | Control |
| BM 1 | 15.27 (0.24) | 12.96 (0.24) |  |  |  |
| DEE adj | 44.17 (0.68) | 38.69 (0.62) | DEE | 45.22 (0.75) | 37.82 (0.69) |
| RER | 0.85 (0.01) | 0.83 (0.01) | Act | 5400 (280) | 2557 (256) |
| DFI adj | 48.49 (1.78) | 41.51 (1.61) | DFI | 50.51 (1.84) | 39.83 (1.68) |
| REE adj | 30.89 (0.65) | 26.53 (0.72) | REE | 31.48 (0.71) | 26.04 (0.65) |

BM 1 = body mass between the ages of 4 to 10 weeks-old [g]; DEE adj = body mass-adjusted daily energy expenditure [kJ/day]; DEE = daily energy expenditure [kJ/day]; RER = respiratory exchange ratio; Act = daily activity, distance moved [m/day]; DFI adj = body mass-adjusted daily food intake [kJ/day]; DFI = daily food intake [kJ/day]; REE adj = body mass-adjusted resting energy expenditure [kJ/day]; REE = resting energy expenditure [kJ/day].

**Table S20.** Phenotypic responses of mice given T4 in the drinking water: Results for mutant homozygous mice carrying Giant panda-specific mutation in DUOX2 gene exposed to **0.5 µg T4 /mL** drinking water: Mixed model test statistics (F (df)) and significance levels (P).

|  | Fixed Effects | |  | |
| --- | --- | --- | --- | --- |
| Trait | Body mass | | Treatment | |
|  | F (df) | P | F (df) | P |
| BM 2 |  |  | 1.84 (1,9) | 0.208 |
| Fat mass | 9.08 (1,8) | 0.017 | 5.12 (1,8) | **0.053** |
| Lean mass | 10.20 (1,8) | 0.013 | 0.72 (1,8) | 0.422 |
| EnAssim |  |  | 0.15 (1,8) | 0.711 |
| DWI | 2.82 (1,8) | 0.132 | 1.43 (1,8) | 0.266 |
| DUP | 9.57 (1,8) | 0.015 | 2.03 (1,8) | 0.192 |
| Kidneys | 137.99 (1.7) | <0.001 | 73.55 (1,7) | **<0.001** |
| Liver | 86.34 (1,7) | <0.001 | 4.36 (1,7) | 0.075 |
| Brain | 1.12 (1,7) | 0.326 | 1.40 (1,7) | 0.275 |
| Spleen | 0.22 (1,7) | 0.652 | 0.66 (1,7) | 0.442 |
| Lungs | 1.17 (1,7) | 0.315 | 2.07 (1,7) | 0.194 |
| Sal Gla | 2.68 (1,7) | 0.146 | 1.26 (1,7) | 0.298 |
| MWAT | 0.61 (1,7) | 0.460 | 0.04 (1,7) | 0.851 |
| SubWAT | 0.29 (1,7) | 0.604 | 0.77 (1,7) | 0.409 |
| Stomach | 7.20 (1,7) | 0.031 | 0.94 (1,7) | 0.366 |
| BAT | 0.18 (1,7) | 0.687 | 17.24 (1,7) | **0.004** |
| EpWAT | 3.13 (1,7) | 0.120 | 0.75 (1,7) | 0.792 |
| RtWAT | 0.79 (1,7) | 0.402 | 0.83 (1,7) | 0.394 |
| Heart | 34.97 (1,7) | 0.001 | 13.68 (1,7) | **0.008** |
| Pancreas | 0.12 (1,7) | 0.738 | 2.76 (1,7) | 0.141 |
| Small Int | 7.83 (1,7) | 0.027 | 5.98 (1,7) | **0.044** |
| Large Int | 0.06 (1,7) | 0.812 | 0.19 (1,7) | 0.680 |
| Caecum | 0.13 (1,7) | 0.913 | 1.67 (1,7) | 0.237 |
| Skin | 13.02 (1,7) | 0.009 | 0.05 (1,7) | 0.832 |
| Tail | 15.15 (1,7) | 0.006 | 0.31 (1,7) | 0.597 |
| T3 |  |  | 12.81 (1,8) | **0.007** |
| T4 |  |  | 19.70 (1,8) | **0.002** |

BM 2 = body mass at 10-11 weeks-old at physiological measurements [g]; Fat mass = body fat mass [%]; Lean mass = body lean mass [%]; EnAssim = energy assimilation [%]; DWI = daily water intake [g]; DUP = daily urine production [g]; Kidneys = mass of kidneys; Liver; Brain; Spleen; Lungs; Sal Gla = salivary gland; MWAT = mesenteric white adipose tissue; SubWAT = subcutaneous white adipose tissue; Lungs; Stomach ; BAT = brown adipose tissue; EpWAT = epididymal white adipose tissue; RtWAT = retroperitoneal white adipose tissue; Heart; Pancreas; Small Int = small intestine; Large Int = large intestine; Caecum; Skin; Tail [g]; T3 = triiodothyronine [nmol/L]; T4 = thyroxine [nmol/L].

**Table S21.** Adjusted means (LSM) of body composition in mutant homozygous mice carrying Giant panda-specific mutation in DUOX2 gene exposed (T4) and non-exposed (Control) to **0.5** **µg T4 /mL** drinking water.

|  | Adjusted means: LSM (SE) | |  | Adjusted means: LSM (SE) | |
| --- | --- | --- | --- | --- | --- |
| Trait | T4 | Control | Trait | T4 | Control |
| BM 2 | 18.57 (1.02) | 16.70 (0.93) | Stomach | 0.155 (0.005) | 0.163 (0.005) |
| Fat mass | 10.80 (1.23) | 6.89 (1.11) | BAT | 0.120 (0.007) | 0.080 (0.007) |
| Lean mass | 84.28 (1.42) | 85.97 (1.28) | EpWAT | 0.257 (0.039) | 0.242 (0.039) |
| EnAssim | 90.72 (0.41) | 90.49 (0.37) | RtWAT | 0.053 (0.015) | 0.073 (0.015) |
| DWI | 3.63 (0.39) | 4.28 (0.35) | Heart | 0.129 (0.002) | 0.128 (0.002) |
| DUP | 0.78 (0.17) | 1.12 (0.16) | Pancreas | 0.184 (0.019) | 0.139 (0.019) |
| Kidneys | 0.316 (0.004) | 0.266 (0.004) | Small Int | 0.775 (0.026) | 0.686 (0.026) |
| Liver | 0.958 (0.021) | 1.020 (0.021) | Large Int | 0.161 (0.011) | 0.155 (0.011) |
| Brain | 0.431 (0.004) | 0.438 (0.004) | Caecum | 0.114 (0.010) | 0.096 (0.010) |
| Spleen | 0.055 (0.004) | 0.059 (0.004) | Skin | 3.255 (0.129) | 3.215 (0.129) |
| Lungs | 0.140 (0.008) | 0.124 (0.008) | Tail | 0.416 (0.010) | 0.424 (0.010) |
| Sal Gla | 0.122 (0.007) | 0.110 (0.007) | T3 | 1.75 (0.27) | 0.37 (0.27) |
| MWAT | 0.188 (0.021) | 0.182 (0.021) | T4 | 139.87 (19.78) | 15.65 (19.78) |
| SubWAT | 0.204 (0.019) | 0.180 (0.019) |  |  |  |

BM 2 = body mass at 10-11 weeks-old at physiological measurements [g]; Fat mass = body fat mass [%]; Lean mass = body lean mass [%]; EnAssim = energy assimilation [%]; DWI = daily water intake [g]; DUP = daily urine production [g]; Kidneys = mass of kidneys; Liver; Brain; Spleen; Lungs; Sal Gla = salivary gland; MWAT = mesenteric white adipose tissue; SubWAT = subcutaneous white adipose tissue; Lungs; Stomach ; BAT = brown adipose tissue; EpWAT = epididymal white adipose tissue; RtWAT = retroperitoneal white adipose tissue; Heart; Pancreas; Small Int = small intestine; Large Int = large intestine; Caecum; Skin; Tail [g]; T3 = triiodothyronine [nmol/L]; T4 = thyroxine [nmol/L].


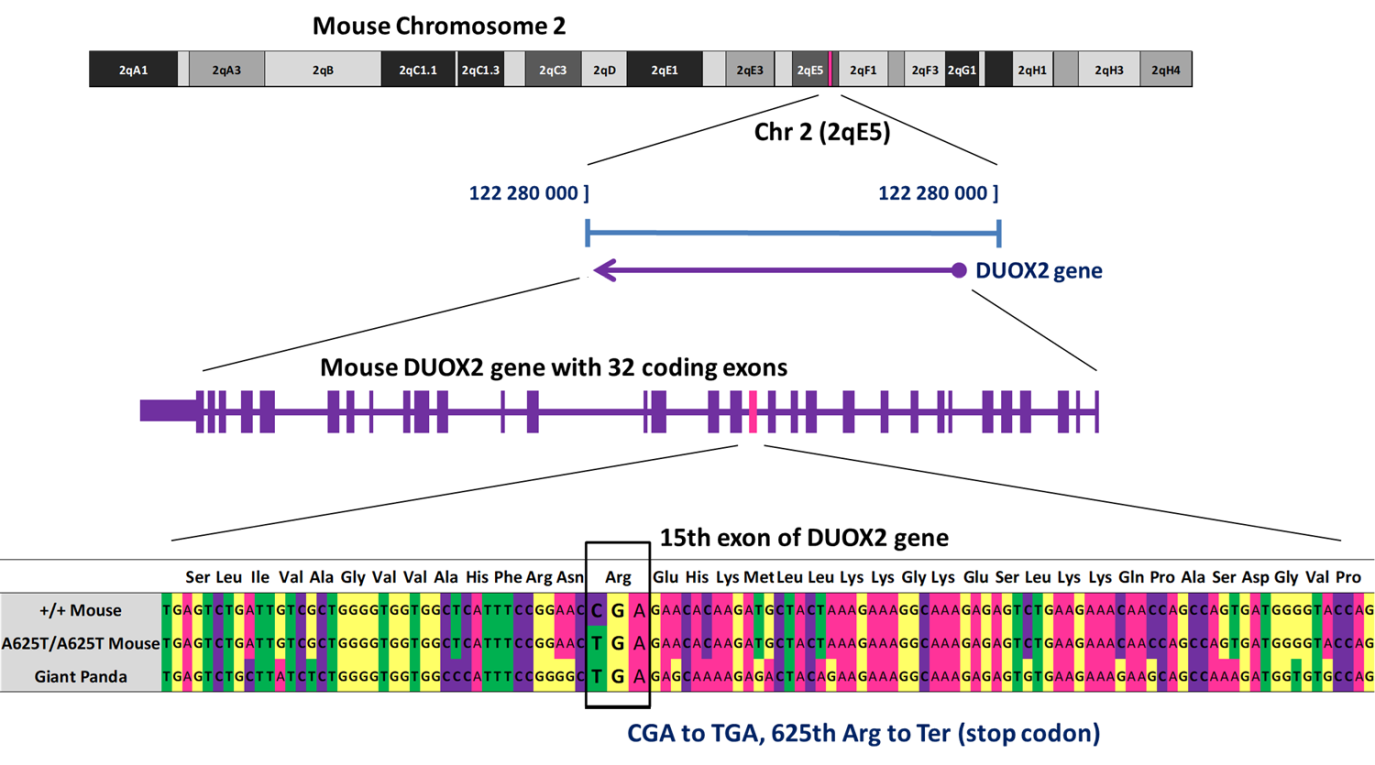


**Figure S1.** The mouse model constructed with conditional strategy Cas9. The wild-type DUOX2^+/+^ and DUOX2^A625T/A625T^ mice are based on C57BL/6J background. At chromosome 2, DUOX2 gene, exon 15th, CGA was changed to TGA, i.e. 625th Arginine to Ter (stop) codon. The mutation is analogous to that found in the giant panda.


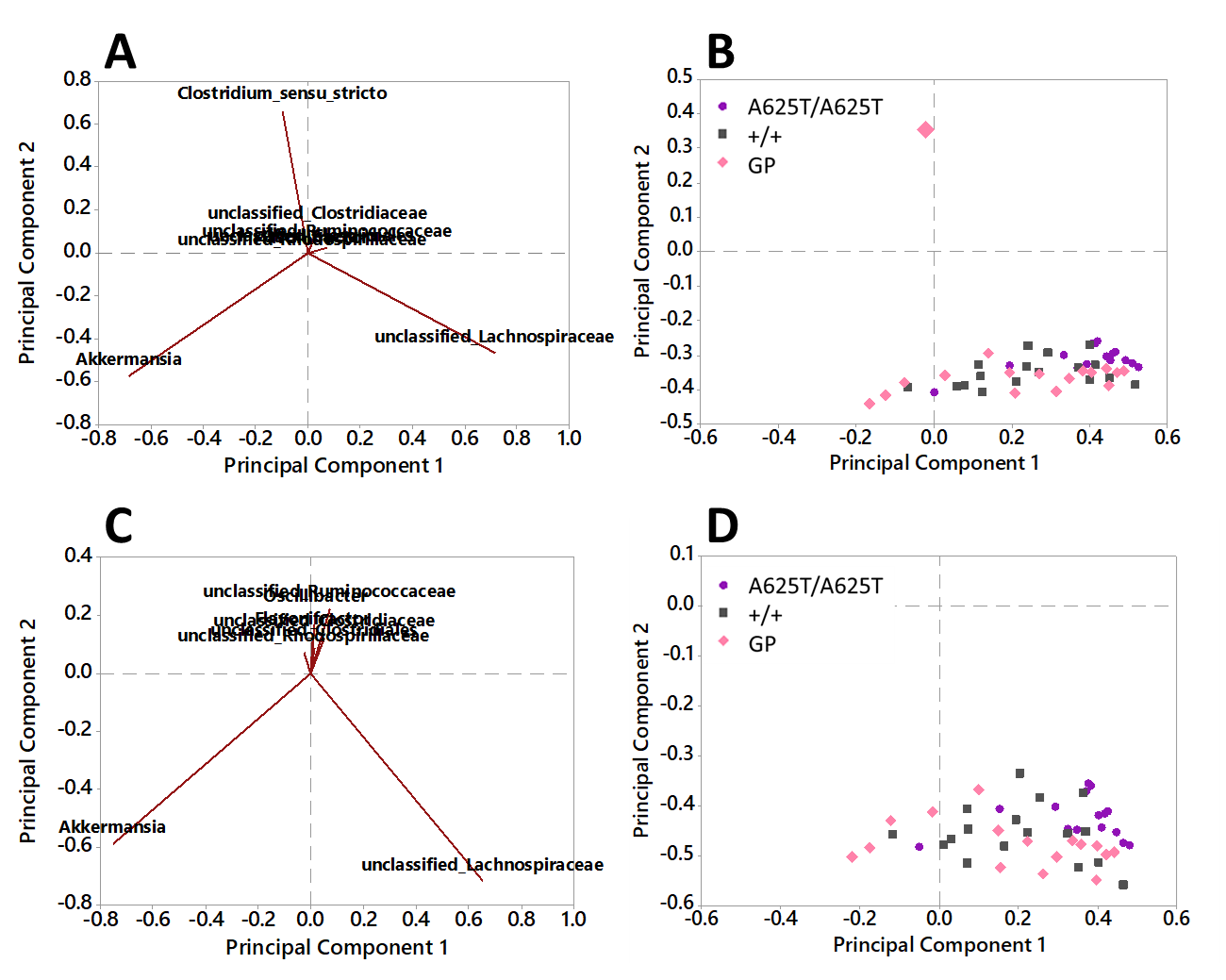


**Figure S2.** Principal Component (PC) analysis of feces microbiota in wild-type mice exposed to microbiota from mutant homozygous (DUOX2^A625T/A625T^), wild-type homozygous (DUOX2^+/+^), or giant panda (GP) donors: (A) PC1 and PC2 with treatment including donor panda sample; (B) PC1 and PC2 with microbiota genus including panda sample; (C) PC1 and PC2 with treatment after excluding donor panda sample; (D) PC1 and PC2 with microbiota genus after excluding panda sample.

**
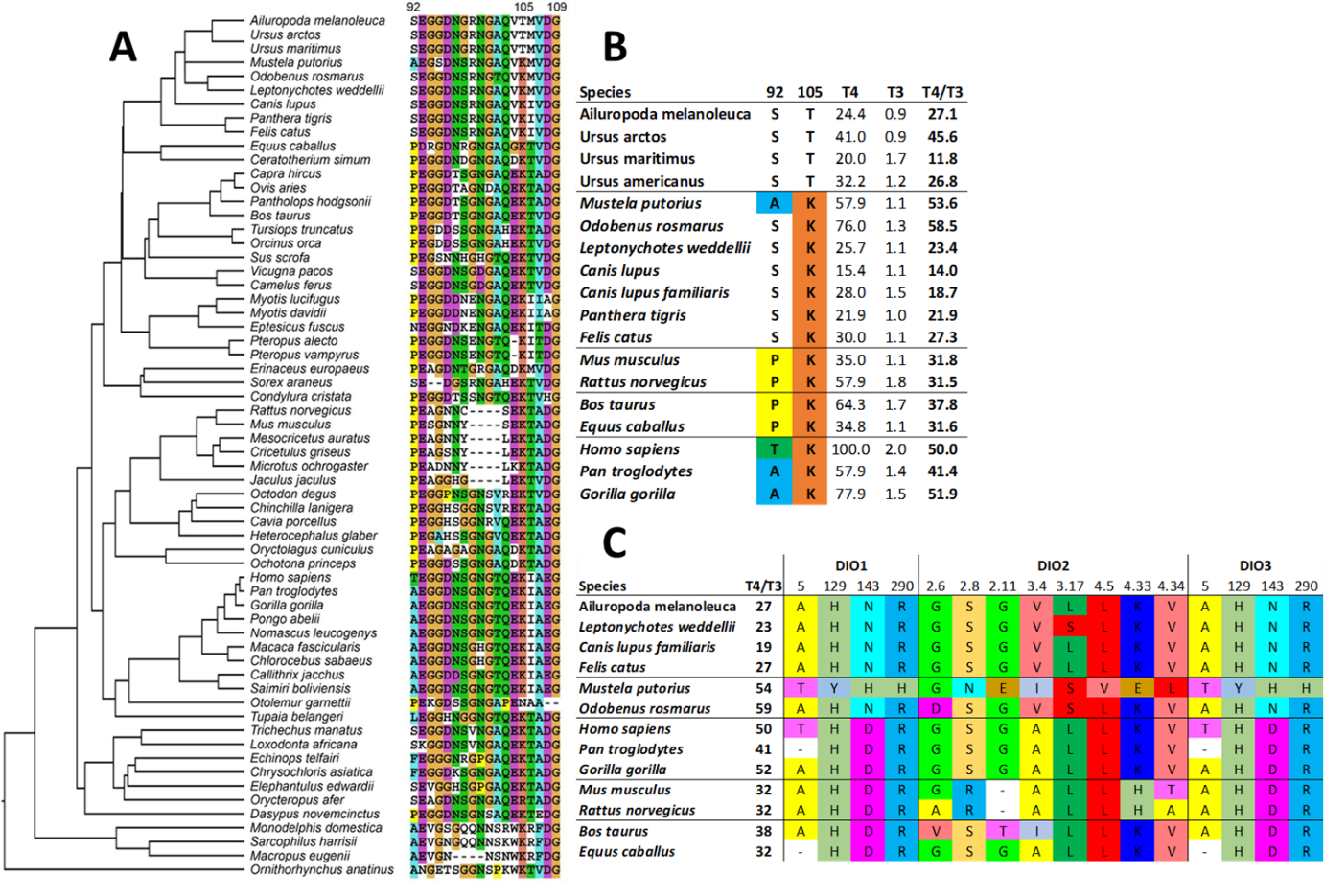
**

**Figure S3.** (A) Amino acid sequence alignment of 18-residue region of DIO2 gene in 62 mammals: high polymorphism at 92nd position, and specific polymorphism at the 105th position present in Ursidae family, (B) T4, T3 [nmol/L], and ratio of T4 to T3 in species carrying similar polymorphisms in 92nd and 105th position of the DIO2, (C) amino acid alignment at 16 positions of DIO1, DIO2 and DIO3 genes in carnivores with low and high T4 to T3 proportion, in primates, rodents and ungulates (exon number of DIO2 before dot).
